# Supplementary material for: Generation of ultra-broadband frequency comb in strongly bistable nonlinear magnonic resonator
Source: Nat Commun. 2026 Jun 13;17:7526. doi: 10.1038/s41467-026-74143-4 (PMC13408486; doi:10.1038/s41467-026-74143-4)
Supplement: Supplementary file 1 — Supplementary Information [file 41467_2026_74143_MOESM1_ESM.pdf]

Supplementary Information for Generation of Ultra-Broadband Frequency Comb in Strongly Bistable Nonlinear Magnonic Resonator

Yu Jiang,[1] Vasyl Tyberkevych,[2] Yizhong Huang,[1] Zixin Yan,[1]
Amin Pishehvar,[1] Andrei Slavin,[2] and Xufeng Zhang[1,3,*]

[1]*Department of Electrical and Computer Engineering, Northeastern University, Boston, MA 02115, USA*
[2]*Department of Physics, Oakland University, Rochester, MI 48309, USA*
[3]*Department of Physics, Northeastern University, Boston, MA 02115, USA*

(Dated: May 14, 2026)

Supplementary Note 1. Theoretical analysis of parametrically assisted excitation of strongly nonlinear magnetization precession

TABLE S1. Key Variables and Parameters

Symbol	Physical Meaning	Definition	Appearance
	**Uniform Magnon Mode**		
a	Normalized complex amplitude of uniform magnon mode		Eq. (1)
N_0	Power of uniform magnon mode	$N_0 = \|a\|^2$	Eq. (S2)
ω_0	Intrinsic FMR frequency		Eq. (1)
$\tilde{\omega}_0$	Nonlinearly shifted FMR frequency	$\tilde{\omega}_0 = \omega_0 + \omega_{\mathrm{M}} N_0 + 2\omega_{\mathrm{M}} N_{\mathrm{p}}$	Eq. (3), Eq. (S6)
Γ	Damping rate of uniform mode		Eq. (1)
	**Spin Wave Modes**		
c_k	Complex amplitude of spin wave mode (with wave vector k)		Eq. (2)
N_{p}	Total parametric magnon power	$N_{\mathrm{p}} = \sum_k \|c_k\|^2$	Eq. (S8)
ω_k	Intrinsic spin wave frequency		Eq. (4)
$\tilde{\omega}_k$	Nonlinearly shifted spin wave frequency	$\tilde{\omega}_k = \omega_k + 2\omega_{\mathrm{M}} N_0 + 2\omega_{\mathrm{M}} N_{\mathrm{p}}$	Eq. (4), Eq. (S7)
Γ_k	Damping rate of spin wave mode	$\Gamma_k = \Gamma$ assumed	Eq. (S5)
	**Drive Parameters**		
ω_1	First pump tone frequency	$\omega_1 = \omega_{\mathrm{s}}$	Main text
ω_2	Second pump tone frequency	$\omega_2 = \omega_{\mathrm{s}} + \delta$, rad/s	Main text
ω_{s}	Nominal pump frequency	$\omega_{\mathrm{s}} = \omega_1$	Eq. (1)
δ	Pump frequency spacing	$\delta = \omega_2 - \omega_1$	Eq. (1)
$\Delta\omega_{\mathrm{s}}$	Pump detuning	$\Delta\omega_{\mathrm{s}} = \omega_{\mathrm{s}} - \omega_0$	Eq. (S2)
F_{s}	Drive amplitude		Eq. (1)
P_{s}	Input signal power	$P_{\mathrm{s}} = \|F_{\mathrm{s}}\|^2$	Eq. (S2)
	**Material Parameters**		
ω_{M}	Nonlinearity coefficient	$\omega_{\mathrm{M}} = \gamma\mu_0 M_{\mathrm{s}}$	Eq. (1)
γ	Gyromagnetic ratio	$\gamma/2\pi = 28$ GHz/T	After Eq. (S1)
M_{s}	Saturation magnetization	$M_{\mathrm{s}} = 140$ kA/m	After Eq. (S1)
	**Threshold Powers**		
N_{par}	Threshold N_0 for parametric instability	$N_{\mathrm{par}} = \Gamma/\omega_{\mathrm{M}}$	Eq. (S10)
P_{par}	Input power for parametric threshold	$P_{\mathrm{par}} \approx \Gamma(\Delta\omega_{\mathrm{s}})^2/\omega_{\mathrm{M}}$,	Eq. (5), Eq. (S11)
P_{min}	Lower bistability boundary		Eq. (S3)
P_{max}	Upper bistability boundary	$P_{\mathrm{max}} \approx (4/27)\Delta\omega_{\mathrm{s}}^3/\omega_{\mathrm{M}}$	Eq. (6), Eq. (S3)

This work considers the reduction of the threshold for excitation of a strongly nonlinear regime of magnetization precession due to parametric interaction of uniform precession mode with plane spin waves.

The specific case examined is that of a normally magnetized thin-film ferromagnetic element driven by a microwave field with frequency ω_{s} significantly higher than the linear ferromagnetic resonance (FMR) frequency ω_0. The important factor is the relative detuning $\frac{\Delta\omega_{\mathrm{s}}}{\Gamma}$, where $\Delta\omega_{\mathrm{s}} = \omega_{\mathrm{s}} - \omega_0$ is the frequency detuning of the driving signal and Γ is the FMR linewidth. Experimentally, values up to $\frac{\Delta\omega_{\mathrm{s}}}{\Gamma} \sim 100$ were investigated and throughout this analysis $\Delta\omega_{\mathrm{s}} \gg \Gamma$ is assumed. The schematic view of the spin wave spectrum in such configuration is shown in Fig. S1.

Direct excitation of strongly nonlinear regime of magnetization precession

The analysis begins with the *direct* excitation of magnetization precession, i.e., without account of the interaction of uniform precession with plane spin waves. This is a standard problem in nonlinear oscillator dynamics, but it is instructive to repeat the main results in order to compare them with the new findings presented here.

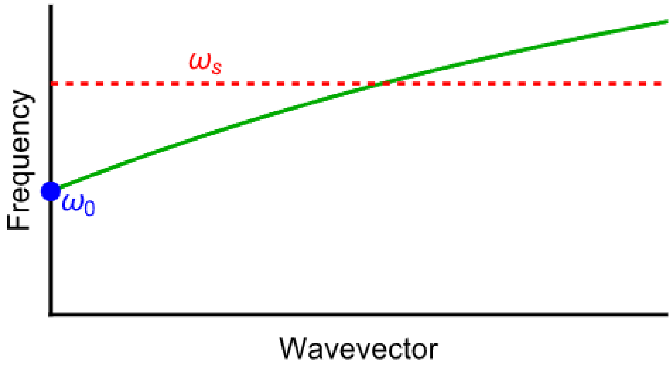

FIG. S1. Schematic view of the spin wave spectrum in normally magnetized thin-film ferromagnetic element. Blue dot shows the FMR frequency. The green line shows the spectrum of plane spin waves. Red dashed line corresponds to the excitation frequency ω_s.

The uniform precession can be described by the complex amplitude $a = \frac{(m_x - i m_y)}{\sqrt{1+m_z}}$ ($\mathbf{m}$ is the unit magnetization vector and z is the normal direction to the film). Its dynamics under external microwave excitation is described by the nonlinear oscillator equation

$$\frac{da}{dt} + \Gamma a = -i \left(\omega_0 + \omega_\mathrm{M} |a|^2\right) a + i F_\mathrm{s} e^{-i\omega_\mathrm{s} t}, \tag{S1}$$

where $\omega_\mathrm{M} = \gamma \mu_0 M_\mathrm{s}$ is the nonlinearity coefficient in normally magnetized geometry (γ is the modulus of gyromagnetic ratio, μ_0 is the vacuum permeability, and M_s is the saturation magnetization; $\omega_\mathrm{M} \approx 2\pi \cdot 4.9\,\mathrm{GHz}$ for YIG at room temperature) and F_s is the amplitude of microwave signal in frequency units. For a linearly polarized microwave magnetic field with magnitude b_s, $F_\mathrm{s} = \frac{\gamma b_\mathrm{s}}{2\sqrt{2}}$.

From Eq. (S1) one can easily derive equation for the stationary oscillator power $N_0 = |a|^2$ of the uniform precession:

$$\left[(\Delta\omega_\mathrm{s} - \omega_\mathrm{M} N_0)^2 + \Gamma^2\right] N_0 = P_\mathrm{s}, \tag{S2}$$

where $P_\mathrm{s} = |F_\mathrm{s}|^2$ is input signal power.

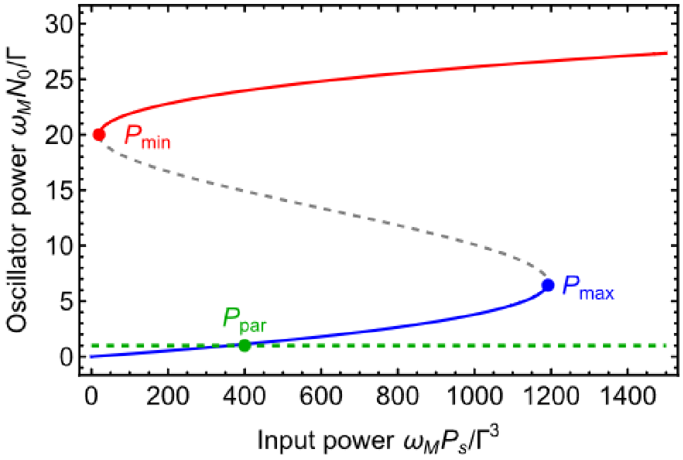

FIG. S2. Stationary oscillator power Eq. (S2) for detuning $\Delta\omega_\mathrm{s} = 20\Gamma$. Blue and red lines show the low- and high-amplitude branches, respectively. Dashed gray line shows the intermediate unstable branch. P_min is the minimum input power for high-amplitude solution. P_max is the maximum input power for which the low-amplitude solution exists. The green dashed line shows the threshold uniform precession power N_par (see Eq. (S10)) for parametric excitation of plane spin waves and P_par is the corresponding input signal power.

The most notable and well-known feature of the stationary solution Eq. (S2) is the bistability – existence of two distinct branches (low-amplitude and high-amplitude) in a certain range of input powers $P_\mathrm{min} < P_\mathrm{s} < P_\mathrm{max}$. This is

illustrated by Fig. S2, where the solution Eq. (S2) is plotted for detuning $\Delta\omega_{\rm s} = 20\Gamma$. The boundaries of the bistable region can be easily found from Eq. (S2) and, for $\Delta\omega_{\rm s}/\Gamma \gg 1$, are given by

$$P_{\rm min} \approx \frac{\Gamma^2 \Delta\omega_{\rm s}}{\omega_{\rm M}}, \qquad P_{\rm max} \approx \frac{4}{27}\frac{\Delta\omega_{\rm s}^3}{\omega_{\rm M}}. \tag{S3}$$

As one can see from Eq. (S3), for large detuning the two critical powers $P_{\rm min}$ and $P_{\rm max}$ are drastically different, $\frac{P_{\rm max}}{P_{\rm min}} \sim \left(\frac{\Delta\omega_{\rm s}}{\Gamma}\right)^2$. Thus, for $\Delta\omega_{\rm s} = 100\Gamma$ they differ by more than 30 dB. For example, considering an experimental conditions with $\Delta\omega_{\rm s} = 100\Gamma = 2\pi \cdot 183$ MHz the lower critical power $P_{\rm min}$ corresponds to the driving signal magnitude $b_{\rm min} \approx 0.036$ mT, which is easily achievable experimentally. On the other hand, the upper critical power $P_{\rm max}$ requires much higher microwave field $b_{\rm max} \approx 1.38$ mT, which is beyond the capabilities of most experimental setups.

This large difference between $P_{\rm min}$ and $P_{\rm max}$ has important consequences for excitation of the strongly nonlinear high-amplitude branch of the magnetization precession. The condition $P_{\rm s} > P_{\rm min}$ can be easily satisfied, thus, it is easy to perform experiments in which the high-amplitude branch exists. However, to directly excite this branch, the instantaneous signal power must exceed $P_{\rm max}$, which, as explained above, is practically impossible. Thus, excitation of the strongly nonlinear magnetization precession requires an additional mechanism that reduces the threshold of excitation. Such mechanism naturally exists in normally magnetized ferromagnetic samples, as explained below.

Parametrically assisted excitation of strongly nonlinear magnetization precession

As one can see in Fig. S1, the excitation frequency $\omega_{\rm s}$ crosses the linear spin wave spectrum at a certain wavevector k determined by the spin wave dispersion law $\omega_k = \omega_{\rm s}$. The plane spin waves with non-zero wavevector k cannot be excited directly by uniform microwave magnetic field; however, these waves can be excited parametrically by uniform precession in so-called second order Suhl process.

Mathematically, this process is described by the system of equations for uniform precession

$$\frac{da}{dt} + \Gamma a = -i\left(\omega_0 + \omega_{\rm M}|a|^2 + 2\omega_{\rm M}\sum_k |c_k|^2\right)a - i\omega_{\rm M}\left(\sum_k c_k c_{-k}\right)a^* + iF_{\rm s}e^{-i\omega_{\rm s}t} \tag{S4}$$

and plane spin waves with wavevector k:

$$\frac{dc_k}{dt} + \Gamma_k c_k = -i\left(\omega_k + 2\omega_{\rm M}|a|^2 + 2\omega_{\rm M}\sum_k |c_k|^2\right)c_k - i\omega_{\rm M}a^2 c^*_{-k}. \tag{S5}$$

In these equations, parametric interaction between plane spin waves is neglected, which does not change the qualitative picture of interaction, and it is assumed that many mutually incoherent spin wave pairs can be excited simultaneously (these pairs have the same magnitude of wavevector $|k|$ but different directions of propagation). In Eq. (S5) Γ_k is the damping rate of spin wave with wavevector k. For simplicity, in the following analysis $\Gamma_k = \Gamma$ is assumed.

Equations (S4)–(S5) describe two important effects. First, there is parametric interaction between uniform precession and plane spin waves, described by the terms $\omega_{\rm M}\left(\sum_k c_k c_{-k}\right)a^*$ in Eq. (S4) and $\omega_{\rm M}a^2c^*_{-k}$ in Eq. (S5). Second, they describe mutual nonlinear frequency shift leading to nonlinear renormalization of the resonant frequency of the uniform precession

$$\widetilde{\omega}_0 = \omega_0 + \omega_{\rm M}N_0 + 2\omega_{\rm M}N_{\rm p}, \tag{S6}$$

and plane spin waves

$$\widetilde{\omega}_k = \omega_k + 2\omega_{\rm M}N_0 + 2\omega_{\rm M}N_{\rm p}, \tag{S7}$$

where $N_{\rm p}$ is the total power of the parametrically excited spin waves:

$$N_{\rm p} = \sum_k |c_k|^2. \tag{S8}$$

The difference in factor of 2 in the term $\omega_{\rm M}N_0$ in Eqs. (S6) and (S7) comes from different efficiencies of nonlinear self- and cross-interaction, but it is not important for the considered effects.

As follows from Eq. (S5), parametric instability of plane spin wave pair with wavevectors develops when power of the uniform precession exceeds threshold value

$$N_0 > \frac{\sqrt{\Gamma^2 + (\widetilde{\omega}_k - \omega_{\mathrm{s}})^2}}{\omega_{\mathrm{M}}}. \tag{S9}$$

The most unstable pairs are at the exact resonance, $\widetilde{\omega}_k = \omega_{\mathrm{s}}$, for which the condition Eq. (S9) simplifies to

$$N_0 > N_{\mathrm{par}} = \frac{\Gamma}{\omega_{\mathrm{M}}}. \tag{S10}$$

For high-quality ferromagnetic materials like YIG N_{par} lies deep in the low-amplitude branch of the nonlinear ferromagnetic resonance curve shown in Fig. S1 (see green line in Fig. S1). The corresponding input signal power can be easily found in the limit $\Delta\omega_{\mathrm{s}} \gg \Gamma$:

$$P_{\mathrm{par}} \approx \frac{\Gamma \Delta\omega_{\mathrm{s}}^2}{\omega_{\mathrm{M}}}. \tag{S11}$$

The power P_{par} is intermediate between the lower P_{min} and upper P_{max} critical powers, as shown in Fig. S1. For $\Delta\omega_{\mathrm{s}} = 100\Gamma$ and experimental parameters, the threshold magnitude of the microwave magnetic field is $b_{\mathrm{par}} = 0.37\,\mathrm{mT}$, which is within the capabilities of our experimental setup.

Thus, for large frequency detunings $\Delta\omega_{\mathrm{s}}$ the parametric excitation of plane spin waves by non-resonantly driven uniform precession starts at reasonably low input power levels, for which the magnetization precession is still within the low-amplitude branch.

To see how the parametric excitation of plane spin wave assists with the excitation of the high-amplitude uniform precession, consider the case of input signal just barely exceeding the threshold value Eq. (S11). In this case the total number of parametric magnons $N_{\mathrm{p}}(t)$ grows very slowly with time and one can use quasi-static analysis with $N_{\mathrm{p}}(t)$ as a slowly varying parameter.

Increasing the magnon number $N_{\mathrm{p}}(t)$ has two effects on dynamics of the uniform precession mode. First, it increases effective damping through the parametric coupling term $\omega_{\mathrm{M}} \left(\sum_k c_k c_{-k}\right) a^*$. This effect is important in the case of resonant excitation ($\Delta\omega_{\mathrm{s}} = 0$) of uniform precession, when its stationary amplitude is limited by damping (in the resonant case $|a| = \frac{|F_{\mathrm{s}}|}{\Gamma}$). In our case of substantially non-resonant excitation ($\Delta\omega_{\mathrm{s}} \gg \Gamma$) the stationary amplitude of uniform precession is not damping-limited ($|a| \approx \frac{|F_{\mathrm{s}}|}{\Delta\omega_{\mathrm{s}}}$) and the increase of effective damping is of minor importance and can be ignored.

Another, much more important effect is the nonlinear frequency shift induced by the parametric magnons (see Eq. (S6)). This frequency shift reduces the effective frequency detuning to

$$\widetilde{\Delta\omega_s} = \Delta\omega_{\mathrm{s}} - 2\omega_{\mathrm{M}} N_{\mathrm{p}}, \tag{S12}$$

and, respectively, reduces the upper critical power $\widetilde{P}_{\mathrm{max}}(N_{\mathrm{p}})$ for transition to the high-amplitude branch of magnetization precession. At a certain level of parametric magnon population $\widetilde{P}_{\mathrm{max}}(N_{\mathrm{p}})$ becomes equal to the actual signal power P_{s} and the uniform precession jumps to the high-amplitude branch. The frequency shift, required for this transition, is

$$2\omega_{\mathrm{M}} N_{\mathrm{p}} = \delta\omega_{\mathrm{th}} = \Delta\omega_{\mathrm{s}} - \left(\frac{27}{4}\omega_{\mathrm{M}} P_{\mathrm{s}}\right)^{\frac{1}{3}}. \tag{S13}$$

It should be noted that parametrically excited magnons also shift up the plane spin wave spectrum $\widetilde{\omega}_k$ (see Eq. (S7)). This shift, however, does not qualitatively modify the process of parametric excitation as long as resonant spin waves $\widetilde{\omega}_k = \omega_{\mathrm{s}}$ exist in the spectrum. The whole spin wave spectrum shifts above the excitation frequency for $2\omega_{\mathrm{M}} N_{\mathrm{p}} = \Delta\omega_{\mathrm{s}}$, which requires higher magnon densities than the threshold Eq. (S13).

To illustrate the process described above, numerical simulations of the dynamics of the spin wave system described by Eqs. (S4)–(S5) were performed. An example of such simulations is shown in Fig. S3 for $\Delta\omega_{\mathrm{s}} = 100\Gamma$ and relatively low input signal power P_{s}, which was only 2 dB above the threshold of parametric excitation P_{par} Eq. (S11) and about 10 dB below the upper critical power P_{max} Eq. (S3).

Figure S3 shows the time dependence of the power of the uniform mode $N_0(t)$. As one can see, it stays for a long time at the low-amplitude stationary value, and then (at $t \approx 3.15\,\mu\mathrm{s}$) rapidly transitions to the high-amplitude

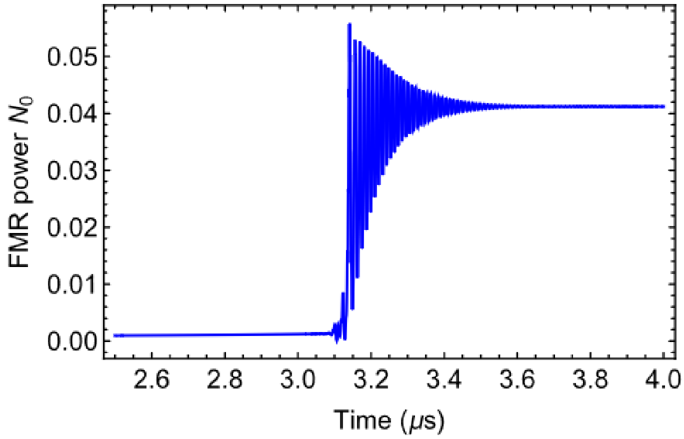

FIG. S3. Time dependence of the power of uniform precession mode $N_0(t)$ in the presence of parametrically excited plane spin waves. Simulation parameters: spin wave damping rate $\Gamma = 2\pi \cdot 1.83\,\mathrm{MHz}$, frequency detuning $\Delta\omega_\mathrm{s} = 100\Gamma = 2\pi \cdot 183\,\mathrm{MHz}$, input signal amplitude $F_\mathrm{s} = 2\pi \cdot 4.45\,\mathrm{MHz}$ (amplitude of the driving magnetic field $b_\mathrm{s} = 0.45\,\mathrm{mT}$).

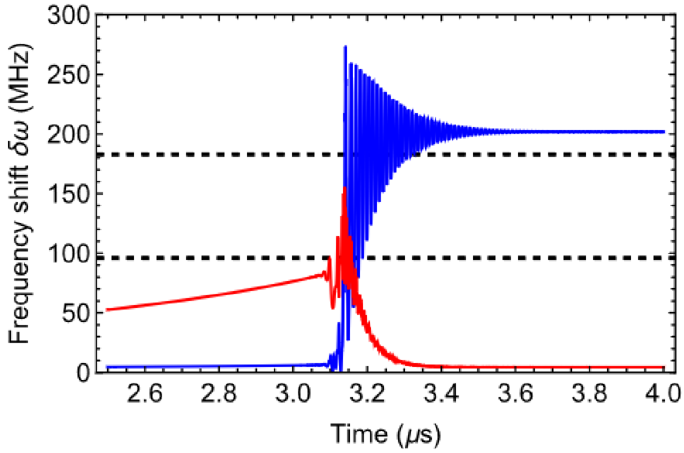

FIG. S4. Time dependence of nonlinear frequency shifts $\delta\omega$. Red line: frequency shift $2\omega_\mathrm{M} N_\mathrm{p}$ due to parametrically excited magnons. Blue line: frequency shift $\omega_\mathrm{M} N_0$ due to uniform precession mode (this line is the same as in Fig. S3, but scaled to frequency units). Dashed horizontal lines show two characteristic values: lower line shows the frequency shift $\delta\omega_\mathrm{th}$ Eq. (S13) required for transition to the high-amplitude branch; upper line shows the frequency detuning $\delta\omega = \Delta\omega_\mathrm{s}$.

branch. Note that such long "dwelling times" (of the order of μs) are due to input power P_s chosen, for illustrative purposes, to be very close to the threshold value P_par and, respectively, very slow growth of parametrically excited magnons.

To gain better insight into the transition process, Fig. S4 shows the time dependence of the nonlinear frequency shift $2\omega_\mathrm{M} N_\mathrm{p}$ induced by parametric magnons (red line in Fig. S4). As can be observed, the number of parametric magnons increases continuously while uniform precession remains on the low-frequency branch. These magnons have minor influence on the dynamics of the uniform precession until the induced frequency shift remains lower than the threshold shift $\delta\omega_\mathrm{th}$ (lower horizontal line in Fig. S4). At this point uniform precession rapidly (within ns time scale) transitions to the upper strongly nonlinear high-amplitude branch.

Thus, the results of numerical simulations of the system Eqs. (S4)–(S5) fully confirm the presented above qualitative picture of parametrically assisted excitation of strongly nonlinear magnetization precession.

To complete the consideration of this effect, we shall consider processes that happen after the uniform precession switched to the high-amplitude branch (region $t > 3.15\,\mu\mathrm{s}$ in Figs. S3 and S4). The decaying oscillations seen in the interval $3.5\,\mu\mathrm{s} > t > 3.15\,\mu\mathrm{s}$ are the usual transient oscillations in the forced dynamics of oscillators. More interesting is the dynamics of the parametrically excited magnons in the high-amplitude regime: as one can see in Fig. S4, the total number of parametric magnons rapidly decay.

This phenomenon is explained by the nonlinear frequency shift $\omega_\mathrm{M} N_0$ induced by the uniform precession. As one

can see, in the high-amplitude regime this frequency shift exceeds the signal detuning $\Delta\omega_{\rm s}$ (upper horizontal line in Fig. S4). Respectively, the whole spin wave spectrum, which is shifted by $2\omega_{\rm M}N_0$ (see Eq. (S7)), moves above the excitation frequency and there are no spin wave modes satisfying the nonlinear resonance condition $\widetilde{\omega}_k = \omega_{\rm s}$. Respectively, the parametric magnon population rapidly decays as seen in Fig. S4.

Thus, the parametric excitation of plane spin waves is important only for the transition from low-amplitude to high-amplitude branches of uniform precession.

Supplementary Note 2. Experimental Details

The SSPP waveguide is designed to have a cutoff frequency around 10 GHz, where the transmission magnitude drops together with a drastic reduction in the group velocity, leading to a rapid phase change. A typical transmission spectrum of our device is shown in Fig. S5, with the magnon mode superimposed on it. Near the SSPP cutoff frequency, the efficiency of the microwave pumping to the magnons is significantly enhanced due to the slow-wave enhancement effect. In fact, due to the small volume of our YIG micro-resonator, the magnon signals cannot be observed when conventional magnon transducers are used, such as microstrips or co-planar waveguides, which make it highly challenging to observe the magnon frequency combs (MFCs).

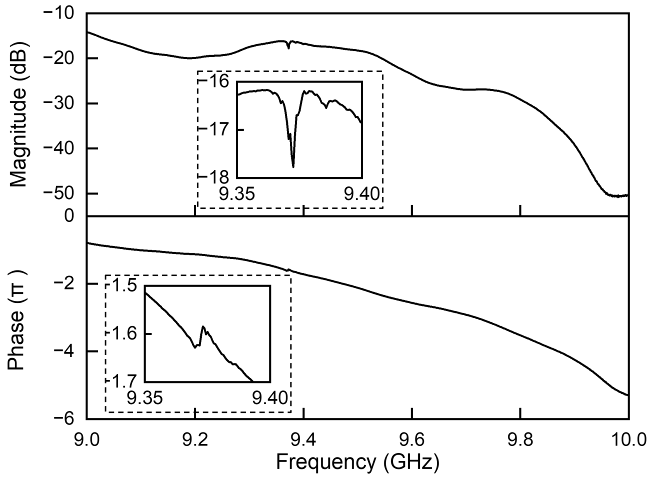

FIG. S5. Transmission spectrum of the SSPP waveguide measured using the VNA near the SSPP cutoff frequency at around 10 GHz. Insets: the magnon resonance.

The measured MFC spectrum on the spectrum analyzer for different bias magnetic fields and pump detunings are plotted in Fig. S6, which are used to extract the data shown in Figs. 3a and 3b of the main text. Note that the powers shown here are the measured power on the spectrum analyzer, which is 20 dB higher than the on-chip power considering the channel loss at the output.

Figure S7 shows a linear-scale plot of the threshold power as a function of the pump detuning $\Delta\omega_s$, showing their quadratic relation.

Figure S9 plots more details scan of the MFC spectra at different pump frequency difference $\delta = \omega_2 - \omega_1$. The y axis is the frequency span, calculated as the actual frequency subtracting 10 GHz. The two horizontal bright lines near the top are the two pumps, with one frequency fixed while the other one scanned nearby. The white region below the two lines are the comb generation regime. Evidently the comb span increases as δ decreases. But when the two pumps are degenerate, the MFC disappears due to the missing amplitude oscillation of the pump power.

Figure S8 shows the homodyne detection setup used to record the time-domain signal of the MFC output. A microwave source at $\omega_{\rm LO} = \omega_1$ is used as the local oscillator (LO), allowing the MFC signal to be down-converted to baseband. The down-converted signal is then passed through a 10 MHz high-pass filter before being digitized by an analog-to-digital converter (ADC). This filtering step suppresses strong near-DC feedthrough from the two pump tones—Pump ω_1 converting to DC and Pump ω_2 converting to $\delta/2\pi < 10$ MHz—thereby revealing the periodic output of the MFC, which would otherwise be obscured by beating between the pumps. An IQ mixer is employed to acquire both the in-phase and quadrature components, and the final time trace is reconstructed from these two channels. A power splitter directs a copy of the signal to a spectrum analyzer for simultaneous spectral measurement. All instrumentation is controlled through a computer-based acquisition system, which collects and organizes the data.

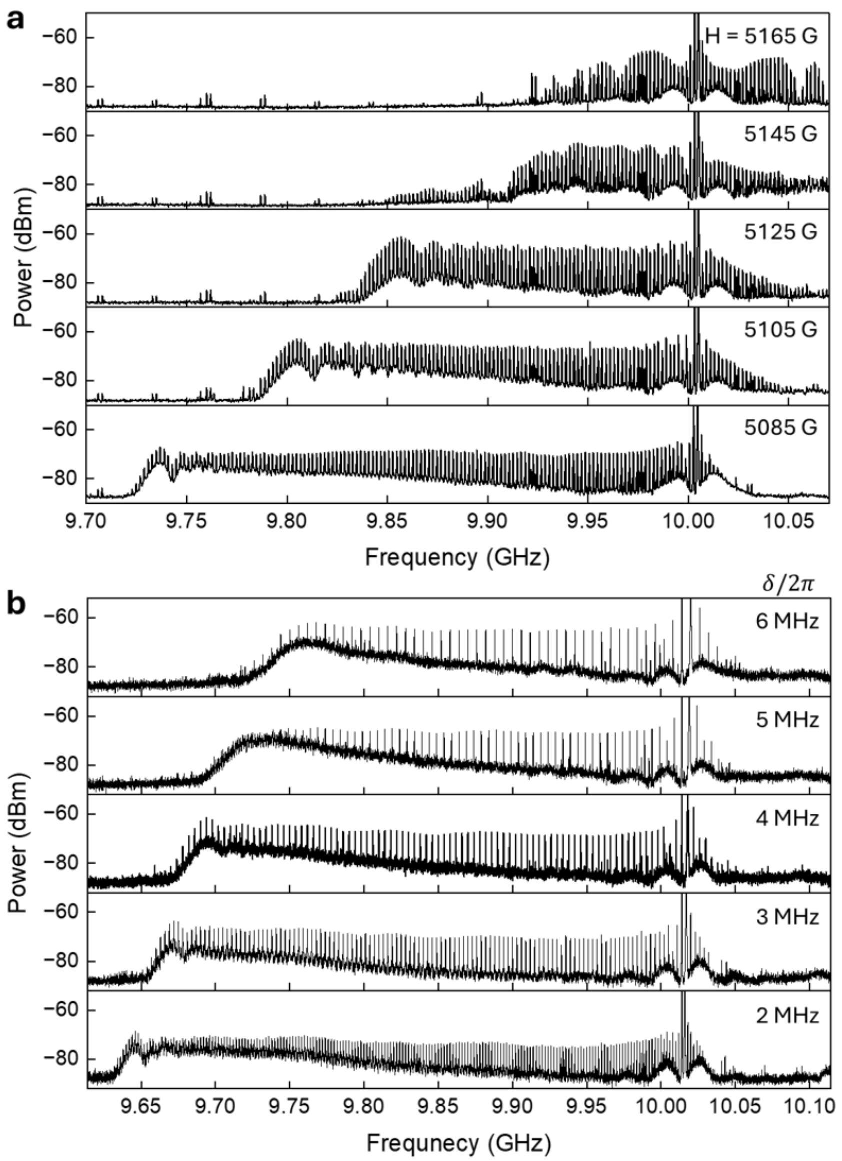

FIG. S6. Device output spectrum at different bias magnetic field and pump detunings $\delta = \omega_2 - \omega_1$.

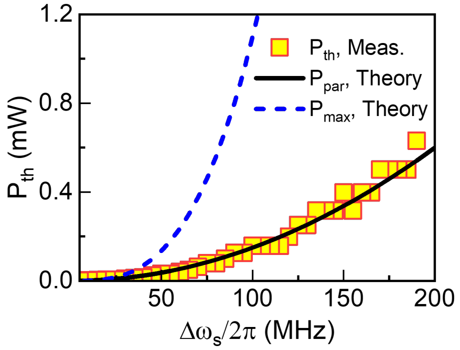

FIG. S7. Linear-scale plot of the threshold power $P_{\rm th}$ as a function of the pump detuning $\Delta\omega_{\rm s}$, together with the theoretical $P_{\rm par}$ and $P_{\rm max}$ values.

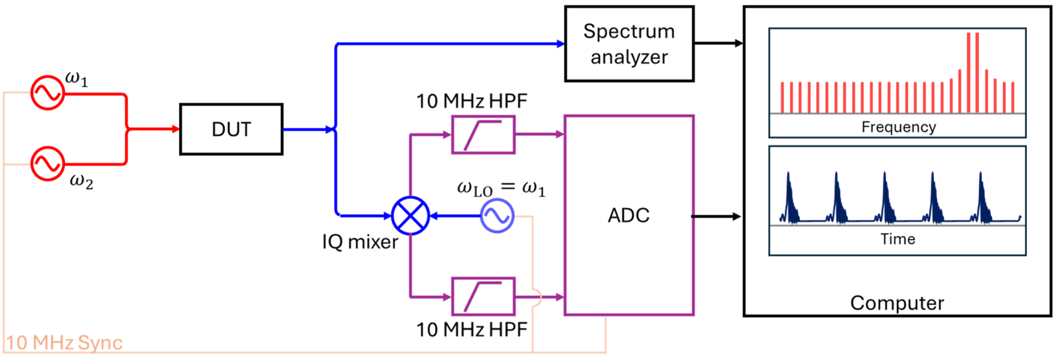

FIG. S8. Measurement scheme for the homodyne detection of the time trace. HPF: high-pass filter; ADC: Analog-to-digital converter; DUT: device-under-test.

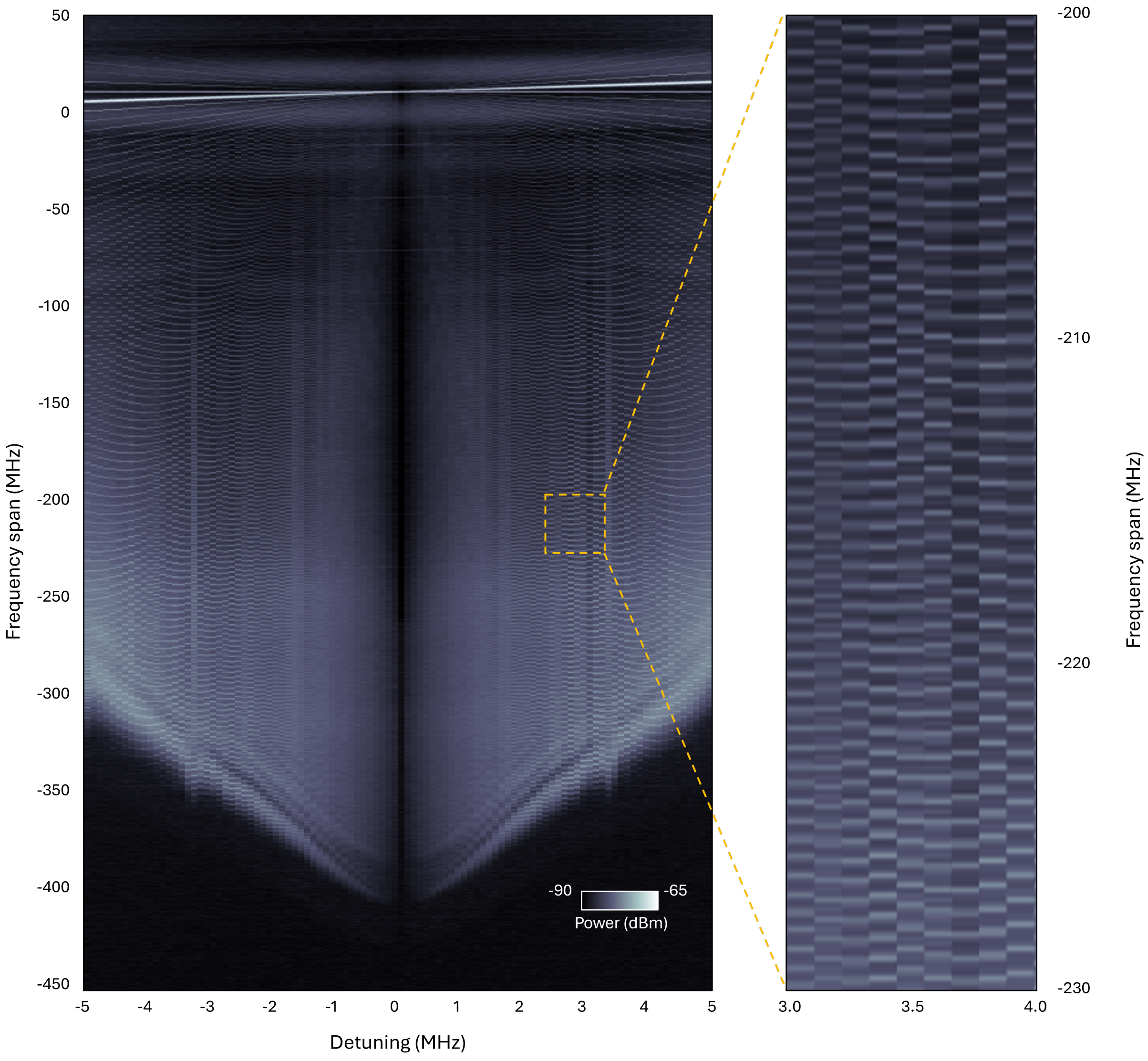

FIG. S9. Device output spectrum at different pump detunings $\delta = \omega_2 - \omega_1$.

Supplementary Note 3. Comparison with the state of the art

Table S2 summarizes the details of the reported MFCs in recent years. These data are used for Fig. 4c of the main text. Both experimental and theoretical results are included. The total comb line count of 350 obtained in this work is the largest among all the previously reported values, even including the theoretical values, regardless of the actual physical platform utilized and frequencies of operation. Compared with the experimentally obtained comb line counts, our results is over one order of magnitude higher and has the potential to further expand.

TABLE S2. State of the art in MFC generation

	**Material**	**Mechanism**	**Line count**	**Comb span**	**Line spacing**	**Pump freq.**	**Reference**
**Experiment**	**on-chip YIG microresonator**	Kerr effect	**350**	450 MHz	1.3 MHz	10 GHz	This work
	YIG sphere	Kerr effect	32	4 MHz	0.1 MHz	3 GHz	Ref. [1]
	YIG sphere	Magnomechanical interaction	20	200 MHz	10 MHz	4.7 GHz	Ref. [2]
	CoFe waveguide	Kerr effect	7	4 GHz	0.5 GHz	10 GHz	Ref. [3]
**Theory**	YIG sphere	Parametric pumping	20	50 MHz	2.5 MHz	10 GHz	Ref. [4]
	YIG sphere	Magnomechanical interaction	23	250 MHz	11.42 MHz	28 GHz	Ref. [5]
	YIG sphere	Magnomechanical interaction	60	660 MHz	11.42 MHz	NA	Ref. [6]
	YIG sphere	Magnomechanical interaction	120	1.37 GHz	11.42 MHz	NA	Ref. [7]
	Permalloy disk	Magnon-vortex interaction	44	8 GHz	0.18 GHz	6.1 GHz	Ref. [8]
	NA	Magnon-Skyrmion interaction	50	50 GHz	1 GHz	50 GHz	Ref. [9]
	NA	Magnon-Skyrmion interaction	25	200 GHz	8 GHz	80 GHz	Ref. [10]
	Co waveguide	Magnon-Skyrmion interaction	20	200 GHz	5.9 GHz	80 GHz	Ref. [11]
	GdCo/Co	Magnon-Skyrmion interaction	9	1 THz	100 GHz	500 GHz	Ref. [12]

References

* xu.zhang@northeastern.edu

[1] C. Wang, J. Rao, Z. Chen, K. Zhao, L. Sun, B. Yao, T. Yu, Y.-P. Wang, and W. Lu, Enhancement of magnonic frequency combs by exceptional points, Nat. Phys. **20**, 1139 (2024).
[2] G.-T. Xu, M. Zhang, Y. Wang, Z. Shen, G.-C. Guo, and C.-H. Dong, Magnonic frequency comb in the magnomechanical resonator, Phys. Rev. Lett. **131**, 243601 (2023).
[3] T. Hula, K. Schultheiss, F. J. T. Gonçalves, L. Körber, M. Bejarano, M. Copus, L. Flacke, L. Liensberger, A. Buzdakov, A. Kákay, M. Weiler, R. Camley, J. Fassbender, and H. Schultheiss, Spin-wave frequency combs, Appl. Phys. Lett. **121**, 112404 (2022).
[4] A. Kani, M. Hatifi, and J. Twamley, Squeezed microwave and magnonic frequency combs, APL Quantum **2**, 016112 (2025).
[5] H. Xiong, Magnonic frequency combs based on the resonantly enhanced magnetostrictive effect, Fundam. Res. **3**, 8 (2023).
[6] Z.-X. Liu, J. Peng, and H. Xiong, Generation of magnonic frequency combs via a two-tone microwave drive, Phys. Rev. A **107**, 053708 (2023).
[7] B. Wang, X.-H. Lu, X. Jia, and H. Xiong, Mechanically induced magnonic frequency combs via the magnetostrictive effect, Phys. Rev. A **111**, 063703 (2025).
[8] Z. Wang, H. Y. Yuan, Y. Cao, and P. Yan, Twisted magnon frequency comb and penrose superradiance, Phys. Rev. Lett. **129**, 107203 (2022).
[9] X. Liang, Y. Cao, P. Yan, and Y. Zhou, Asymmetric magnon frequency comb, Nano Lett. **24**, 6730 (2024).
[10] Z. Wang, H. Y. Yuan, Y. Cao, Z.-X. Li, R. A. Duine, and P. Yan, Magnonic frequency comb through nonlinear magnon-skyrmion scattering, Phys. Rev. Lett. **127**, 037202 (2021).
[11] J. Sun, S. Shi, and J. Wang, Strain modulation of magnonic frequency comb by magnon–skyrmion interaction in ferromagnetic materials, Adv. Eng. Mater. **24**, 2101245 (2022).
[12] Y. Liu, T. Liu, Q. Yang, G. Tian, Z. Hou, D. Chen, Z. Fan, M. Zeng, X. Lu, X. Gao, M. Qin, and J. Liu, Design of controllable magnon frequency comb in synthetic ferrimagnets, Phys. Rev. B **109**, 174412 (2024).
