# Supplementary material for: Generation of ultra-broadband frequency comb in strongly bistable nonlinear magnonic resonator
Source: Nat Commun. 2026 Jun 13;17:7526. doi: 10.1038/s41467-026-74143-4 (PMC13408486; doi:10.1038/s41467-026-74143-4)
Supplement: Supplementary file 2 — Transparent Peer Review file [file 41467_2026_74143_MOESM2_ESM.pdf]

nature portfolio

Peer Review File

Generation of Ultra-Broadband Frequency Comb in Strongly Bistable Nonlinear Magnonic Resonator

Corresponding Author: Professor Xufeng Zhang

This file contains all reviewer reports in order by version, followed by all author rebuttals in order by version.

Version 0:

Reviewer comments:

Reviewer #1

(Remarks to the Author)
Manuscript ID: NCOMMS-25-100036-T
Title: Generation of Ultra-Broadband Frequency Comb in Strongly Bistable Nonlinear Magnonic Resonator
Authors: Xufeng Zhang, Yu Jiang, Vasil Tiberkevich, Yizhong Huang, Zixin Yan, Amin Pishehvar, and Andrei Slavin

In the submitted manuscript (MS), the authors experimentally present a novel approach to realize ultra-broadband magnonic frequency combs (MFCs) by engineering the nonlinear response of a nonlinear magnonic YIG-film microresonator directly driven by a two-tone microwave pump field consisting of two frequencies. Notably, they report over 350 comb lines spanning a 450 MHz bandwidth with continuously tunable spacing via two-tone driving, achieving an order-of-magnitude enhancement compared to prior work while requiring relatively low power consumption. Featuring an ultra-compact footprint (4-6 orders of magnitude smaller than traditional YIG sphere resonators), full scalability, and high tunability, this platform enables precise manipulation of comb properties through tailored magnetic biasing and pump tuning.

While this work reported here contains interesting experimental observations that contribute to the next-generation functional devices driven by nonlinear magnetic phenomena, after careful evaluation, there are concerns and questions that need to be addressed. As some of these issues are potentially critical to the MS's foundation, I cannot recommend the MS until the authors provide clarifications. Below are my specific concerns:

1. The nonlinear interaction between the uniform magnon mode and parametrically excited spin waves in YIG under transverse pumping has been previously investigated, where the frequency comb-like phenomenon, period doubling, and chaos were observed experimentally, see for example Ref. [PRA, 29, 811 (1984)]. For comparative purposes, could the authors please elaborate on both the core differences between the present work and those relevant studies? This comparison would help readers understand the incremental advances of the present research.

2. To theoretically interpret the key experimental results (ultrabroadband MFC generation) presented in this work, Eqs. (1)-(2) seem to serve as the foundational framework. Could the authors please perform a theoretical simulation starting from Eqs. (1)-(2) to generate corresponding physical results, which can then be directly compared with the experimental findings presented in this work? This quantitative comparison between the simulated outcomes and experimental data would significantly strengthen the theoretical validation of the observed phenomena.

3. To generate ultrabroadband MFCs, it seems that the nonlinear coupling coefficient ω_M in Eqs. (1)-(2) emerges as a critical parameter. Could the authors please further explain how the coupling coefficient was derived, as well as the factors that influence its value?

4. As the measured MFC spectrum shown in Figs. (3e), (4d) and (4e), the signal intensity at the pump frequency is remarkably strong, which indicates that the energy efficiency of the generated MFCs is relatively low. Could the authors please explain the underlying reasons for this phenomenon and provide insights into potential methods to improve the energy conversion efficiency?

5. There are a few typographical errors in the MS, and here are some examples below.
1) The symbols for the mode frequencies of the uniform magnon mode and the spin-wave modes appear to be different in

the main text (line 112) compared to the supplementary file [Eqs. (S6)-(S7)].
2) Line 35 in the supplementary file: the symbol “Ω_s” may be a typo and should be “Δω_s”.
3) For the references, the abbreviation of journal names should be consistent. Currently, many journal names are not abbreviated, see Refs. [1], [6], [8-12], etc.

Reviewer #2

(Remarks to the Author)
The authors demonstrate the generation of a magnonic frequency comb (MFC) with a high line count and an broadband spectral range in a micron-scale magnetic thin-film device, utilizing strong nonlinearity. Unlike conventional on-chip frequency combs (e.g., optical combs) that typically rely on a series of equidistant physical modes in a multi-mode resonator, the authors employ a miniaturized YIG resonator coupled with a slow-wave waveguide. This approach successfully achieves an efficient MFC generation mechanism, yielding a bandwidth of 450 MHz and over 350 comb lines.

A key aspect of their implementation is the use of large detuning conditions, which creates a state with significant magnon amplitude disparity. This appears to be crucial for the effective modulation of magnon amplitude. The utilization of large detuning is a clever strategy, providing valuable insights for readers on leveraging bistability for efficient frequency comb generation.

Overall, the authors present a novel and effective method for MFC generation. I find the work interesting and potentially suitable for publication. However, I have several specific questions and comments that need to be addressed to clarify the physical mechanisms and data presentation.

Specific Comments:
(1)The authors emphasize the importance of "large detuning." Could the authors clarify what specific detuning condition is most favorable for MFC generation? For instance, in the schematic of Fig. 1c, why is the detuning not increased further to reach the peak of the curve? Would this yield a larger dynamic modulation range of the amplitude, or are there other limiting factors (e.g., power handling or stability)?

(2)In Fig. 1d, the reader learns that the hysteresis loop transitions are defined by P_{min} and P_{par}. Therefore, in Fig. 2a, the dashed line indicating P_{max} seems somewhat redundant and potentially distracting, as the system triggers at P_{par}. I suggest removing the P_{max} line in Fig. 2a to focus the reader's attention on the actual switching thresholds (P_{par} and P_{min}).

(3)The authors state that parametric excitation reduces the hysteresis threshold, thereby lowering the threshold for MFC generation. This is a significant claim. However, the main text primarily relies on indirect calculation results and schematics to explain this. The manuscript lacks a direct comparison under conditions where parametric excitation is "on" vs. "off". I encourage the authors to provide at least a quantitative assessment (via simulation or estimation) to demonstrate the magnitude of the threshold reduction caused by parametric excitation. This would help readers better appreciate its contribution and have a clearer mind of how to manipulate the threshold of comb generation.

(4)Following the previous point, the high line count and broadband nature of the reported MFC rely on a combination of factors: strong nonlinearity, parametric excitation, bistable response, and the slow-wave waveguide. Could the authors discuss which of these factors is the dominant contributor to enhancing the number of comb lines and expanding the bandwidth? Clarifying these contributions would add significant physical depth to the discussion.

(5)The volume of the YIG film is extremely small ($0.2 \times 50 \times 50 \mu m^3$), yet the pump power in some experiments approaches 0 dBm (nearly mW). In such confined geometries, thermal effects in YIG are typically non-negligible. How have the authors accounted for the thermal frequency shift? Could the authors provide evidence or discussion on how thermal effects influence the stability of the MFC signals, and how they distinguish the magnetic nonlinearity from thermal bistability?

(6)The inset in Fig. 3g uses a log-log scale. I am curious why a linear scale was not used to directly visualize the quadratic relationship. I suggest adding a linear-scale plot (perhaps in the Supplementary Information) to ensure the quadratic fit is robust across the entire range.

Minor Corrections:
(7)Fig. 4c: In the vertical axis label, "Comb line cout" is a typo.

(8)Page 5, Right Column: There is a typo "conresponding".

(9)Fig. 3g: The caption states "blue curve shows quadratic fitting," but the curve in the figure appears black or is difficult to distinguish as blue. Please ensure the color matches the description.

Recommendation:
If the authors can clearly address the questions above and provide the necessary clarifications, I would consider recommending this manuscript for publication.

Version 1:

Reviewer comments:

Reviewer #1

(Remarks to the Author)
The authors have answered very well the questions and clarifications asked by the Reviewers. I now recommend this improved manuscript for publication in Nature Communications.

Reviewer #2

(Remarks to the Author)
The authors have satisfactorily addressed all the questions and comments raised in the previous review round. The revisions have strengthened the clarity of the manuscript. I recommend the revised manuscript for publication.

Open Access This Peer Review File is licensed under a Creative Commons Attribution 4.0 International License, which permits use, sharing, adaptation, distribution and reproduction in any medium or format, as long as you give appropriate credit to the original author(s) and the source, provide a link to the Creative Commons license, and indicate if changes were made.
In cases where reviewers are anonymous, credit should be given to 'Anonymous Referee' and the source.
The images or other third party material in this Peer Review File are included in the article’s Creative Commons license, unless indicated otherwise in a credit line to the material. If material is not included in the article’s Creative Commons license and your intended use is not permitted by statutory regulation or exceeds the permitted use, you will need to obtain permission directly from the copyright holder.
To view a copy of this license, visit https://creativecommons.org/licenses/by/4.0/

Responses to Referee #1

In the submitted manuscript (MS), the authors experimentally present a novel approach to realize ultra-broadband magnonic frequency combs (MFCs) by engineering the nonlinear response of a nonlinear magnonic YIG-film microresonator directly driven by a two-tone microwave pump field consisting of two frequencies. Notably, they report over 350 comb lines spanning a 450 MHz bandwidth with continuously tunable spacing via two-tone driving, achieving an order-of-magnitude enhancement compared to prior work while requiring relatively low power consumption. Featuring an ultra-compact footprint (4-6 orders of magnitude smaller than traditional YIG sphere resonators), full scalability, and high tunability, this platform enables precise manipulation of comb properties through tailored magnetic biasing and pump tuning.

While this work reported here contains interesting experimental observations that contribute to the next-generation functional devices driven by nonlinear magnetic phenomena, after careful evaluation, there are concerns and questions that need to be addressed. As some of these issues are potentially critical to the MS's foundation, I cannot recommend the MS until the authors provide clarifications.

Reply: We thank Referee #1 for finding our work interesting, and we are providing clarifications below requested by Referee #1.

Below are my specific concerns:

1. The nonlinear interaction between the uniform magnon mode and parametrically excited spin waves in YIG under transverse pumping has been previously investigated, where the frequency comb-like phenomenon, period doubling, and chaos were observed experimentally, see for example Ref. [PRA, 29, 811 (1984)]. For comparative purposes, could the authors please elaborate on both the core differences between the present work and those relevant studies? This comparison would help readers understand the incremental advances of the present research.

Reply: We thank the Referee for this insightful comment, and explain below the fundamental differences between our current manuscript and the previous studies on nonlinear magnon dynamics, including Ref. [PRA, 29, 811 (1984)].

First of all, the above cited Ref. [PRA, 29, 811 (1984)] explores the **transition to chaos** via parametric instabilities under ***resonant*** transverse pumping, while our work focuses on a **deterministic, non-chaotic switching regime** enabled by a fundamentally different physical approach. The core differences and their physical significance of our current work, compared to previous studies (including Ref. [PRA, 29, 811 (1984)]) are summarized below:

- **Non-resonant bifurcation vs. resonant excitation:** Unlike the resonant driving used in traditional studies, we employ **strongly off-resonance excitation**. This allows us to exploit the nonlinear fold-over of the resonance curve, creating two distinct, well-defined dynamical states (states of low- and high-amplitude precession) with a robust hysteretic transition. This bi-stability is the foundation of the controlled switching and generation of a frequency comb that we report.
- **Deterministic control of magnetization dynamics via two-tone driving:** While previous studies often observed spontaneous or stochastic transitions to chaos, our two-tone excitation (equivalent to a deep amplitude modulation) acts as a deterministic clock. It periodically and

predictably cycles the system between the two dynamical regimes, forcing abrupt and repeatable transitional dynamics that is not present in single-tone, steady-state experiments.

- **Suppression of chaos through spectral engineering:** Another critical distinction lies in our use of normally magnetized thin films. In bulk samples (see e. g. the above cited Ref. [PRA, 29, 811 (1984)]) or film geometries with in-plane magnetization, high-amplitude precession typically triggers a cascade of parametric spin-wave instabilities, leading to the chaotic behavior. In our geometry, the spin-wave spectrum **is shifted far above the excitation frequency** in the high-amplitude regime. This “spectral gap” effectively forbids the parametric decay of the uniform mode, suppressing the transition to chaos and maintaining the stability of the large-amplitude state and deterministic character of the magnetization dynamics.

We have updated the manuscript to clearly show these points by adding the following paragraph (at the bottom of the right column of Page 1):

> …and, therefore, exhibits fixed comb spacing. *It should be noted that our current MFC generation scheme differs fundamentally in its underlying mechanism from the previous studies of spin wave parametric instabilities (see, for example, Ref. [PRA, 29, 811 (1984)]). Previous studies mainly focus on the transition to chaos via parametric instabilities under a resonant transverse pumping, while this work focuses on the deterministic, non-chaotic switching regime enabled by a fundamentally different physical approach. Under this approach the studied magnetic resonator operates in a far-off-resonance regime under a strong two-tone drive, and relies on parametric excitation and suppression of spin waves. As a result, it enables the deterministic generation of MFCs with significantly enhanced performance.* This on-chip MFC platform …

2. To theoretically interpret the key experimental results (ultrabroadband MFC generation) presented in this work, Eqs. (1)-(2) seem to serve as the foundational framework. Could the authors please perform a theoretical simulation starting from Eqs. (1)-(2) to generate corresponding physical results, which can then be directly compared with the experimental findings presented in this work? This quantitative comparison between the simulated outcomes and experimental data would significantly strengthen the theoretical validation of the observed phenomena.

Reply: We agree with the Referee that quantitative validation is essential for strengthening the connection between our theoretical model and the experimental observations. In response to these comments, we have first added the explicit analytical expressions (see the texts below) for the power thresholds governing MFC generation—both with and without parametric spin-wave excitation—directly into the main text. These expressions were previously available only in the Supplementary Materials; their inclusion in the main manuscript now makes the theoretical framework clearer and ensures that all subsequent comparisons are fully transparent.

Here is the material added to the new version of the main text of the manuscript (prior to the section entitled “Dynamics of Ultra-broadband Frequency Comb Generation”):

> *Using Eqs.~(1) and (2), it is possible to obtain an explicit expression for the threshold power P_{th}=P_{par} of the MFC formation [see Eq.~(S11) in the Supplementary Information], expressed as a function of the detuning* $\Delta\omega_s$
>
> $$P_{th} = P_{par} = \frac{\Gamma}{\omega_M} (\Delta\omega_s)^2 \,. \qquad (5)$$

In the case when there is no excitation of plane spin waves (e.g. in the case of small detuning when $\Delta\omega_s \sim \Gamma$) the threshold of the MFC excitation is much larger, and has the following form (see Eq. (S3) in Supplementary materials) :

$$P_{max} = \frac{4}{27\omega_M} (\Delta\omega_s)^3 . \qquad (6).$$

Thus, the reduction of the power threshold of the MFC generation due to the excitation of plane spin waves can be evaluated as $P_{par}/P_{max} = 27\Gamma/4\pi\Delta\omega_s$, which in our experimental case (when $\Delta\omega_s \sim 100\ \Gamma$) is around 0.07. For such device parameters, the effective pump-field amplitude is about 1.4 mT based on Eq.6, which is difficult to achieve experimentally, whereas Eq. 5 corresponds to an accessible pump-field amplitude of only 0.37 mT, which is comfortably within the experimentally accessible microwave drive range.

Building on this clarification, we would also like to emphasize that the theoretical results presented in our manuscript—both the time-domain traces and the corresponding spectra in Fig. 2—were obtained through direct numerical integration of Eqs. (1)–(2). These simulations were performed specifically to test and validate the physical mechanism underlying ultrabroadband MFC generation. The resulting agreement between theory and experiment is multi-point and comprehensive, as summarized below:

1. **Spectral bandwidth**. The simulated comb spectrum in Fig. 2e spans approximately 500 MHz, in close agreement with the experimentally measured bandwidth of ~450 MHz in Fig. 4d.
2. **Threshold scaling**. The inset of updated Fig. 3i shows quantitative agreement between the theoretically predicted and experimentally measured quadratic dependence of the MFC generation threshold on the detuning $\Delta\omega_s = \omega_s - \omega_0$.
3. **Time-domain dynamics**. To further validate the model, we conducted additional time-domain measurements using homodyne detection. After suppressing the direct feedthrough (near DC in the homodyne measurement) with a 10 MHz high-pass filter, we directly captured the temporal envelope of the MFC. The measured trace (in updated Fig. 3e, also shown below) shows a train of equally spaced pulses, while the zoom-in view (updated Fig. 3f) reveals the intra-pulse oscillation structure, which match closely with the simulated dynamics in Fig. 2b, providing direct evidence of their agreement.

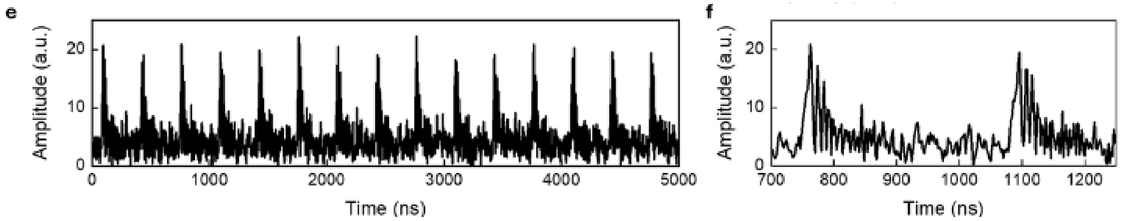

The above presented comparison between theory and experiment demonstrates that the theoretical framework based on Eqs. (1)–(2) successfully reproduces all the physically significant features of the observed MFC dynamics across the spectral, threshold, and time-domain dimensions. This multi-point consistency provides strong support for the validity of our model.

Finally, we note that although full-scale micromagnetic simulations could, in principle, offer additional points of comparison, carrying them out for the parameter ranges relevant to this work would be computationally prohibitively expensive. Such simulations would require resolving all the microscopic details of the system—including thermal fluctuations, short-wavelength parametric instabilities, and the full spatial mode structure of a finite-size magnetic element—which collectively place them far beyond feasible computational limits for the present study. More importantly, the existing level of agreement

between theory and experiment is already extensive and direct, and fully substantiates the mechanisms and interpretations presented in the manuscript.

To better assist the reader, we added the following statement describing the comparison of theory and experiment to the main text of the manuscript (in section Discussion and Outlook):

> *Our theoretical framework, based on direct numerical integration of Eqs. (1)–(2), shows excellent agreement with the experimentally observed dynamics of ultrabroadband MFC generation. The model reproduces all key features of the measured behavior—including the multi-hundred-megahertz comb bandwidth, the quadratic threshold scaling with detuning, and the time-domain pulse-train structure—demonstrating a coherent and self-consistent physical picture. This multi-point correspondence across spectral, threshold, and temporal domains provides strong validation of the underlying mechanism, and confirms that the essential physics of MFC formation is captured by the above presented theoretical description.*

3. To generate ultrabroadband MFCs, it seems that the nonlinear coupling coefficient ω_M in Eqs. (1)-(2) emerges as a critical parameter. Could the authors please further explain how the coupling coefficient was derived, as well as the factors that influence its value?

Reply: The main reason for the nonlinearity in a perpendicularly magnetized magnetic YIG resonator (see Fig. 1a) used in our experiments is the fact that the total length of the magnetization vector in the resonator is conserved, and the parametric excitation of plane spin waves in the resonator leads to the decrease of static magnetization. This effect is mainly determined by the dipole-dipole interaction, and leads both to the nonlinear frequency shift of the main FMR mode in the perpendicularly magnetized YIG resonator (see e. g. equations (20) and (40) in Ref. [IEEE Transactions on Magnetics v.41 p.1264 (2005)]), and to the nonlinear coupling between the uniform FMR mode and parametrically excited plane spin waves. In our model, both the coefficient of the nonlinear frequency shift and nonlinear coupling coefficient are estimated as omega_M.

The coupling coefficient was derived analytically from the Landau-Lifshitz equation using a weakly nonlinear expansion (Holshtein-Primakoff-like approach) in terms of spin-wave amplitudes. We made several simplifying assumptions, which are justified for our specific experimental geometry (a perpendicularly magnetized thin YIG film):

- Negligible ellipticity: In the normal magnetization geometry, the precession of the magnetization is nearly circular. This significantly simplifies the nonlinear terms compared to in-plane magnetized samples, where ellipticity plays a dominant role in determining nonlinear coupling strengths.
- Dominance of dipolar interaction: Because the excited spin waves are close to the bottom of the spin-wave spectrum (long-wavelength limit), the contribution of the exchange interaction to the nonlinear coupling is minimal and was safely neglected.
- Plane-wave approximation: The main assumption in deriving coupling coefficient omega_M is that the excitations can be treated as plane spin waves. Under this approximation, the nonlinear coupling becomes independent of the wavenumber, collapsing into the constant omega_M.

In a real finite-sized sample the nonlinear coupling coefficient will be different, mostly due to spatial inhomogeneity of spin wave modes, but it will always remain of the order of omega_M due to the dominant influence of thin-film-type demagnetization effects.

We estimate that the finite-size effects could shift the effective coupling coefficient by, at most, a factor of two for completely laterally pinned modes. However, we emphasize that such a shift would primarily scale the quantitative value of thresholds, rather than alter the qualitative physics or the existence of the frequency comb. The plane-wave approximation captures the essential physics required to explain the ultrabroadband nature of the MFC while maintaining a transparent analytical framework.

In the main text (in the paragraph under Eq.4) of the revised manuscript, we have added the following text:

> *… The coefficient \omega_M represents both the nonlinear frequency-shift coefficient of the lowest (uniform) magnon mode in our perpendicularly magnetized YIG resonator (see Eq.~(40) in Ref. [IEEE Transactions on Magnetics v.41 p.1264 (2005)]) and its nonlinear coupling to the parametrically excited plane-wave magnons. For simplicity---and without loss of generality---we assume that the excited magnon modes are plane spin waves, allowing \omega_M to be treated as a constant independent of wavenumber and spatial mode profiles. Incorporating realistic mode profiles would refine the quantitative values but does not alter the underlying nonlinear interaction mechanism.*

4. As the measured MFC spectrum shown in Figs. (3e), (4d) and (4e), the signal intensity at the pump frequency is remarkably strong, which indicates that the energy efficiency of the generated MFCs is relatively low. Could the authors please explain the underlying reasons for this phenomenon and provide insights into potential methods to improve the energy conversion efficiency?

Reply: The Referee raises an important point regarding energy efficiency. We would like to address this by distinguishing between the **intrinsic nonlinear conversion efficiency** of the magnetic dynamics and the **extrinsic coupling efficiency** of our experimental setup.

1. **Intrinsic efficiency:** While the spectral density at the pump frequency appears dominant on a logarithmic scale, it is important to consider the integrated power of the entire comb. Our numerical simulations indicate that the primary comb lines are approximately 20 dB above the secondary lines. Given that the generated MFC consists of over 100 lines, the total power contained in the comb spectrum is actually of the same order of magnitude as the power in the driven harmonics. This suggests that once the nonlinear threshold is reached, the energy transfer from the drive to the broader spectrum is quite efficient at the material level.

2. **Experimental efficiency:** The observed low absolute power in our experimental spectra is primarily an artifact of the impedance and mode mismatch between our single magnetic element and the driving circuitry. Namely, the magnetic sample is significantly smaller than the electromagnetic mode volume of the SSPP waveguide used for excitation and detection. Consequently, only a small fraction of the incident microwave power is absorbed by the sample and can be converted into the MFC spectrum, while the rest of the input power becomes direct feedthrough in the transmitted signal and dominates the output spectrum.

To enhance the energy conversion efficiency for practical applications, one may replace the single magnetic element with a synchronized array of nano-elements, which would increase the total interaction volume and the power of the output signal.

To explain the reason of the relatively low energy efficiency of our experimental device for the reader, we added the following text to section “Discussion and outlook” of the revised manuscript:

The relatively low absolute power observed in the experimental spectra is mainly attributable to impedance and mode mismatch between the small magnetic element and the microwave resonator used for excitation. Because the YIG film occupies only a small fraction of the resonator mode volume, only a limited portion of the incident microwave energy is converted into comb power. This limitation is not fundamental: replacing the single element with a synchronized array of magnetic nano-elements would increase the interaction volume and substantially enhance the output power of the comb lines, improving suitability for practical applications.

5. There are a few typographical errors in the MS, and here are some examples below.

1) The symbols for the mode frequencies of the uniform magnon mode and the spin-wave modes appear to be different in the main text (line 112) compared to the supplementary file [Eqs. (S6)-(S7)].

2) Line 35 in the supplementary file: the symbol "Ω_s" may be a typo and should be "Δω_s".

3) For the references, the abbreviation of journal names should be consistent. Currently, many journal names are not abbreviated, see Refs. [1], [6], [8-12], etc.

Reply: We thank Referee #1 for noticing these typographical issues. We have carefully checked the entire manuscript, and corrected all the typos and format issues.

Specifically, we have carefully checked all the mathematical symbols and ensured that the same notation is used in the main text and the Supplementary Information file.

Responses to Referee #2

The authors demonstrate the generation of a magnonic frequency comb (MFC) with a high line count and an broadband spectral range in a micron-scale magnetic thin-film device, utilizing strong nonlinearity. Unlike conventional on-chip frequency combs (e.g., optical combs) that typically rely on a series of equidistant physical modes in a multi-mode resonator, the authors employ a miniaturized YIG resonator coupled with a slow-wave waveguide. This approach successfully achieves an efficient MFC generation mechanism, yielding a bandwidth of 450 MHz and over 350 comb lines.

A key aspect of their implementation is the use of large detuning conditions, which creates a state with significant magnon amplitude disparity. This appears to be crucial for the effective modulation of magnon amplitude. The utilization of large detuning is a clever strategy, providing valuable insights for readers on leveraging bistability for efficient frequency comb generation.

Overall, the authors present a novel and effective method for MFC generation. I find the work interesting and potentially suitable for publication. However, I have several specific questions and comments that need to be addressed to clarify the physical mechanisms and data presentation.

Reply: We thank the Reviwer#2 for stating that our approach to the MFC generation is novel and effective, and evaluating our work as *interesting and potentially suitable for publication.*

We have addressed below all the specific questions and comments of Referee #2:

Specific Comments:

(1) The authors emphasize the importance of "large detuning." Could the authors clarify what specific detuning condition is most favorable for MFC generation? For instance, in the schematic of Fig. 1c, why is the detuning not increased further to reach the peak of the curve? Would this yield a larger dynamic modulation range of the amplitude, or are there other limiting factors (e.g., power handling or stability)?

Reply: We appreciate the Referee's request for clarification on the optimal detuning conditions.

This is a critical parameter that balances the spectral richness of the MFC against the energy required to sustain it. In principle, larger detuning is more favorable for maximizing the MFC bandwidth. The number of generated comb lines is approximately proportional to the ratio of the frequency detuning to the modulation frequency δ. Consequently, increasing the detuning allows for a broader MFC. However, as the Referee correctly suspected, we cannot increase the detuning indefinitely due to the power handling issues. Namely, the threshold power required to reach the bistable "switching" region scales quadratically with the detuning (see Eq.(5) and Fig.4e). Our choice of a maximum detuning of 450 MHz was a practical compromise dictated by the power handling limits of our microwave amplifiers, and the need to avoid excessive Joule heating in the sample.

(2) In Fig. 1d, the reader learns that the hysteresis loop transitions are defined by P_{min} and P_{par}. Therefore, in Fig. 2a , the dashed line indicating P_{max} seems somewhat redundant and potentially distracting, as the system triggers at P_{par}. I suggest removing the P_{max} line in Fig. 2a to focus the reader's attention on the actual switching thresholds (P_{par} and P_{min}).

Reply: We also thank Referee #2 for his insightful comments on our Fig. 1 and Fig. 2.

Regarding Fig.1d: In the original version of the manuscript, Fig. 1c was intended as a conceptual aid, and only used a detuning-to-damping ratio of around 4. However, in our experiments, the ratio of detuning to the damping rate Δ/Γ exceeds 100, which would result in a hysteretic "tongue" so long and narrow that it would be visually difficult to interpret in a standard plot.

In the revised manuscript, we have added to Fig.1 one additional frame (the new Fig.1d, as shown below) with a detuning-to-damping ratio of approximately 100. This frame more accurately reflects the experimental conditions, thus being clearer and more instructive for the reader.

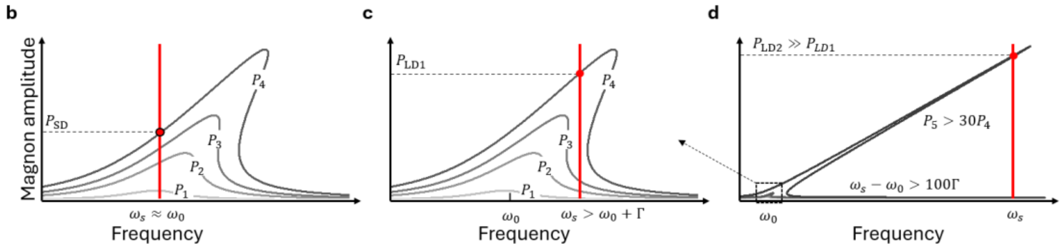

Regarding Fig.2a: We would prefer, however, to retain the P_{max} notation everywhere else in the text, because P_{max} is the threshold power corresponding to the system jump to the upper branch (and formation of the MFC) in **the case when *spin waves are not excited*** , e.g. in the case of small detuning when $\Delta \sim \Gamma$ (see explanations below). So keeping P_{max} would help highlight the importance of parametric spin wave excitation.

(3) The authors state that parametric excitation reduces the hysteresis threshold, thereby lowering the threshold for MFC generation. This is a significant claim. However, the main text primarily relies on indirect calculation results and schematics to explain this. The manuscript lacks a direct comparison under conditions where parametric excitation is "on" vs. "off". I encourage the authors to provide at least a quantitative assessment (via simulation or estimation) to demonstrate the magnitude of the threshold reduction caused by parametric excitation. This would help readers better appreciate its contribution and have a clearer mind of how to manipulate the threshold of comb generation.

Reply: We thank Referee #2 for this insightful comment. The reduction of the MFC excitation threshold via parametric excitation is indeed a central aspect of our work, and we agree that a direct quantitative comparison strengthens the manuscript.

In the original version, the analytical expressions for the power threshold $P_{\rm par}$(applicable when parametric excitation of spin waves occurs) and the threshold $P_{\rm max}$(corresponding to the case without parametric excitation of plane spin waves) were provided only in the Supplementary Information. To clarify this point, the revised manuscript now presents both expressions explicitly in Eqs. (4) and (5), enabling the reader to evaluate the threshold reduction $P_{\rm par}/P_{\rm max} = 27\Gamma/4\pi\Delta\omega_{\rm s}$, which in our experimental regime (where $\Delta\omega_{\rm s} \sim 100\Gamma$) is approximately 0.07.

Without the assistance of parametric excitation, reaching the high-amplitude dynamical regime would require microwave powers far exceeding the maximum output available from our RF source and amplifiers, and/or exceed the handling limits of our device and likely result in sample damage, making such operation

experimentally inaccessible. To provide the direct comparison encouraged by the Referee, we have added to the inset of Fig. 3g the thresholds obtained without spin-wave excitation (in log-log scale. The linear scale plot is provided in the Supplementary Information), plotted as a function of the detuning $\Delta\omega_s$, allowing a direct comparison with the original case where parametric spin-wave excitation is included. This visualization clearly shows that the observed switching follows the P_{par}scaling and occurs at power levels substantially lower than those predicted by the standard nonlinear foldover model.

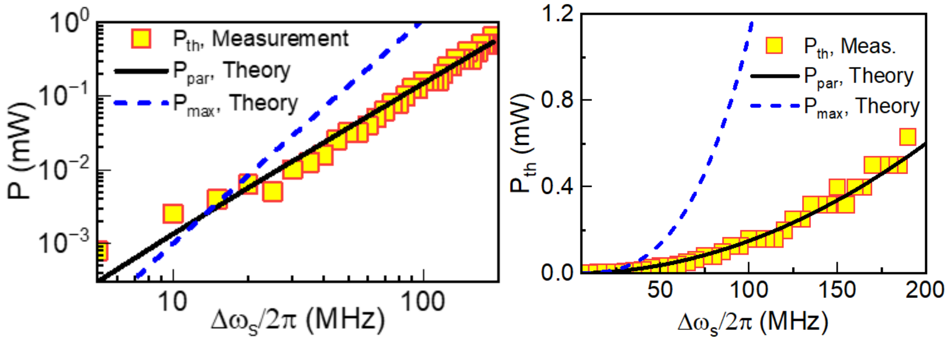

(4) Following the previous point, the high line count and broadband nature of the reported MFC rely on a combination of factors: strong nonlinearity, parametric excitation, bistable response, and the slow-wave waveguide. Could the authors discuss which of these factors is the dominant contributor to enhancing the number of comb lines and expanding the bandwidth? Clarifying these contributions would add significant physical depth to the discussion.

Reply: The dominant contributors to both the large comb-line count and the broadband nature of the reported MFC are the strong nonlinearity of the system and the associated bistable response.

These two factors fundamentally govern the efficiency of energy transfer across widely separated frequency components. In addition, operating at **large frequency detuning is essential**, as it further enhances the accessible bandwidth and enables excitation of high-order comb lines that would otherwise remain inactive.

Parametric excitation, while crucial experimentally, mainly serves to lower the threshold power and make comb generation observable under practical laboratory conditions. It is, therefore, an enabling mechanism rather than a fundamental limitation on the achievable bandwidth or line count.

Similarly, the slow-wave enhancement provided by the waveguide functions as an engineering strategy to supply sufficient pump power density. Its role is analogous to that of the parametric drive: it facilitates observation of the comb but is not the primary factor determining the ultimate extent of the comb.

We have added the following paragraph in the "Discussion and Outlook" section of the revised manuscript to clarify these ideas:

> *The large comb-line count and broadband spectral extent originate primarily from the strong intrinsic nonlinearity of the YIG microresonator and the associated bistable response, which together facilitate efficient energy transfer across widely separated frequency components. Operation at large frequency detuning further broadens the accessible bandwidth by activating high-order comb lines. In contrast, parametric excitation and slow-wave enhancement act chiefly*

as enabling mechanisms that reduce the threshold and increase pump power density, rather than fundamentally determining the achievable comb span.

(5) The volume of the YIG film is extremely small ($0.2 \times 50 \times 50 \mu m^3$), yet the pump power in some experiments approaches 0 dBm (nearly mW). In such confined geometries, thermal effects in YIG are typically non-negligible. How have the authors accounted for the thermal frequency shift? Could the authors provide evidence or discussion on how thermal effects influence the stability of the MFC signals, and how they distinguish the magnetic nonlinearity from thermal bistability?

Reply: We thank the Referee for raising this important point. To assess the influence of heating, we performed additional measurements in which the pump power was further increased using a 35 dB RF amplifier. The resulting data (shown below) reveals that the lower bound of the MFC spectrum (dotted blue line)—corresponding to the intrinsic FMR frequency—shifts upward as the pump power increases. Notably, once the input power exceeds approximately –2 dBm, this upward shift becomes more pronounced. Such a trend is consistent with the thermal effect in a normally magnetized YIG thin film, where heating reduces the demagnetization field, and, consequently, leads to an increase in the FMR frequency.

These observations indicate that thermal shifts occur on timescales much slower than the fast magnon dynamics responsible for MFC generation, and they become significant only at power levels well above those used in our experiments. Within the operational power range of the reported measurements, thermal contributions are minimal, and do not affect the stability or the underlying mechanism of the MFC. For this reason, the thermal effects were not included in the main analysis. We agree, however, that a detailed investigation of thermal behavior will be important for future practical implementations of ultra-wide-band MFCs, which is beyond the scope of this work and will be explored in future works.

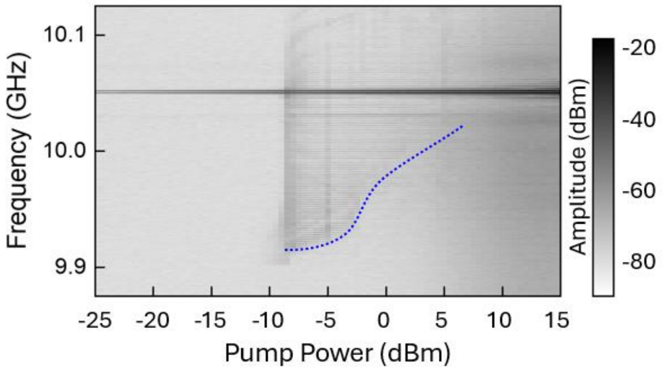

(6) The inset in Fig. 3g uses a log-log scale. I am curious why a linear scale was not used to directly visualize the quadratic relationship. I suggest adding a linear-scale plot (perhaps in the Supplementary Information) to ensure the quadratic fit is robust across the entire range.

Reply: We thank Referee #2 for this helpful suggestion. The inset in Fig. 3g is presented on a log–log scale to clearly demonstrate that the quadratic fit is robust over the full dynamic range; on a linear scale, the agreement at small x and y values become difficult to discern. Nevertheless, following the Referee's

recommendation, we have added a linear-scale version of the plot (see below) to the Supplementary Information, which directly visualizes the quadratic relationship and confirms the robustness of the fit.

Minor Corrections:

(7)Fig. 4c: In the vertical axis label, "Comb line cout" is a typo.

(8)Page 5, Right Column: There is a typo "conresponding".

(9)Fig. 3g: The caption states "blue curve shows quadratic fitting," but the curve in the figure appears black or is difficult to distinguish as blue. Please ensure the color matches the description.

Reply: We thank Referee#2 for noticing these types and errors. We have carefully checked the new version of the manuscript and addressed all of them, and other similar issues.

Recommendation:

If the authors can clearly address the questions above and provide the necessary clarifications, I would consider recommending this manuscript for publication.

Reply: We sincerely believe we have addressed all the concerns of both Referees listed above.

We would like to thank both Referees for the attentive reading of our manuscript, and for their useful and insightful comments.
